# Advancements in two-dimensional nanomaterials for regenerative medicine in skeletal muscle repair

**DOI:** 10.1016/j.mtbio.2025.101924

**Published:** 2025-06-02

**Authors:** Hongyu Bai, Lu Liu, Zhiwen Luo, Renwen Wan, Jiwu Chen

**Affiliations:** a Department of Sports Medicine, Shanghai General Hospital, Shanghai Jiao Tong University, Shanghai, 200080, China; b Department of Sports Medicine, Huashan Hospital, Fudan University, Shanghai, 200040, China

**Keywords:** Two-dimensional nanomaterials, Skeletal muscle repair, Regenerative medicine

## Abstract

Skeletal muscle, the largest organ in the human body, plays vital roles in movement, heat generation, and internal organ protection. While healthy muscle can regenerate effectively, its regenerative capacity declines in conditions like congenital muscular dystrophy, severe trauma, or aging. Two-dimensional (2D) nanomaterials, with unique physicochemical properties such as high surface area, excellent biocompatibility, and tunable mechanical and electrical properties, have shown great promise in different forms of muscle injury, particularly in volumetric muscle loss (VML). Recent studies highlight their diverse applications in muscle regeneration, acting as cell recruitment platforms, drug delivery carriers, structural scaffolds, and anti-inflammatory agents. Additionally, their biological effects and intelligent responsiveness are emerging as key features. Despite these advances, safety concerns regarding toxicity and biodegradability remain a challenge for clinical application. To unlock the full potential of 2D materials, further research is needed, especially through interdisciplinary collaboration to better understand their biological effects. By addressing safety issues and harnessing their multifunctional and intelligent characteristics, 2D nanomaterials can offer a more effective and sustainable approach to skeletal muscle repair, paving the way for next-generation therapies in regenerative medicine.

## Highlights

• 2D nanomaterials promote muscle regeneration by recruiting cells, delivering drugs, and more.
• Advantages in muscle repair include high surface area, biocompatibility, and tunable properties.
• Major limitation for clinical use: safety concerns, especially toxicity and biodegradability.
• Multifunctionality and intelligent responsiveness drive 2D nanomaterial innovation in biomedicine.

## Graphical abstract

**Figure:**
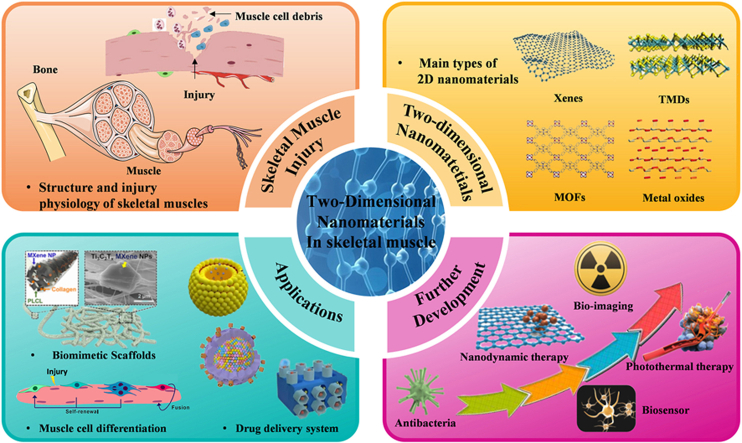

## 1 Introduction

Skeletal muscle, accounting for over 40 % of body weight, is the body's largest organ and is essential for movement, thermogenesis, organ protection and energy homeostasis[[1], [2], [3]]. While healthy adult muscle can typically regenerate after acute injury [4,5], this capacity declines in conditions such as muscular dystrophies, volumetric muscle loss (VML), cachexia, and aging. Tissue fibrosis with chronic inflammation further exacerbates muscle wasting, followed by functional impairment [6].

Studies have shown that the decline in skeletal muscle mass and function is closely associated with an increased risk of morbidity—particularly chronic diseases—and all-cause mortality [7]. Enhancing muscle repair and regeneration is therefore a critical clinical goal.

The discovery of satellite cells and advancements in tissue engineering technologies have deepened our understanding of muscle regeneration principles and methodologies [8]. Over the past two decades, researchers have proposed innovative strategies such as biomimetic scaffolds to simulate muscle microstructure, guiding cellular directional differentiation through biochemical signaling gradient design, and optimizing regenerative microenvironments with physical stimuli (e.g., electrical pulses or mechanical loading) [9,10]. Throughout these developments, functional biomaterials, especially nanomaterials have played systemic roles by providing all-encompassing support for cellular activities, delivering bioactive signals, and dynamically regulating microenvironmental cues to coordinate muscle repair processes [11].

Nano-sized materials or nanomaterials can be defined as substances that have at least one dimension that is less than 100 nm in size [12]. Based on their size and shape, nanomaterials exhibit distinct physicochemical properties compared to bulk materials that are composed of the same elements. Depending on the shape and size of nano-sized dimensions, nanomaterials can be classified into four dimensionality types (zero to three dimensional nanomaterials, compared to other types, two-dimensional (2D) nanomaterials gaining particular attention due to their high surface area, mechanical strength, and tunable bioactivity. These features make 2D nanomaterials especially promising for biomedical applications, including skeletal muscle repair, where they can interact with cells at the nanoscale, modulate the local microenvironment, and enhance tissue regeneration.

In this narrative review, we searched PubMed and Web of Science using the keywords ‘low-dimensional nanomaterials' and ‘muscle’ (from inception to March 19, 2025), and manually selected relevant articles based on their relevance to the topic. We focus on recent studies leveraging 2D nanomaterials for regeneration of skeletal muscle. We summarize representative proof-of-concept and preclinical studies, highlighting how material dimensionality contributes to enhanced regenerative outcomes. Finally, we address remaining scientific challenges and propose optimization strategies to enhance the translational potential of 2D nanomaterial-based therapies.

## 2 Skeletal muscle injury

### 2.1 Skeletal Muscle Anatomy and function

Skeletal muscle is a highly organized tissue composed of muscle fibers arranged into fascicles, with varying lengths and tightly packed in a complementary manner [13]. The fibers are multinucleated and contain myofibrils, which are organized into sarcomeres, the contractile units responsible for muscle function [14]. Skeletal muscle contraction is under voluntary control, making it a ‘voluntary muscle’. The contraction is characterized by rapid and powerful but short-lived actions. Muscle performance is influenced by different fiber types: Type I fibers, known as ‘slow-twitch fibers,’ have higher vascularization and metabolic capacity compared to Type II fibers, or ‘fast-twitch fibers,’ which are adapted for quick, forceful contractions [15].

As a highly vascularized and innervated voluntary muscle, skeletal muscle is essential for postural control, organ protection, locomotion, and other energy-dependent physiological functions driven by muscle fiber contraction [16]. Skeletal muscle also functions as a potential ‘endocrine organ’, communicating with other tissues and organs through the orchestrated release of an array of muscle-derived signaling molecules or myokines, including CCL2, IGF-1, IL-6, IL-8, IL-10, IL-15, IL-1 receptor antagonist, irisin, and myostatin [17,18], etc. These factors are involved in exercise-induced metabolic adaptations, such as glucose regulation [19], lipid homeostasis [20], and increases in muscle mass and vascularization [21].

Additionally, the extracellular matrix (ECM) of skeletal muscles contributes significantly to muscle development, growth, and repair. The composition and structure of ECM can be influenced by training, disease, and aging, thus altering its physiological functions [22].

### 2.2 Classification, mechanisms, and treatments of skeletal muscle injury

Under normal conditions, skeletal muscle continuously adapts to changes in its mechanical environment by modulating gene expression and protein stability. When mechanical stress exceeds its adaptive capacity, acute injury occurs. Common causes of acute skeletal muscle injury include extreme temperatures, contusions, tears, and toxins, which also represent common classifications of muscle damage.

Acute skeletal muscle injury induces inflammation, edema, and other pathological changes, leading to muscle atrophy, a reduction in muscle fiber count, and fat infiltration. These factors, if persistent, further exacerbate widespread muscle necrosis, fibrosis, and severe damage to the microstructure and function of muscle bundles [23,24].

**Fig. 1:**
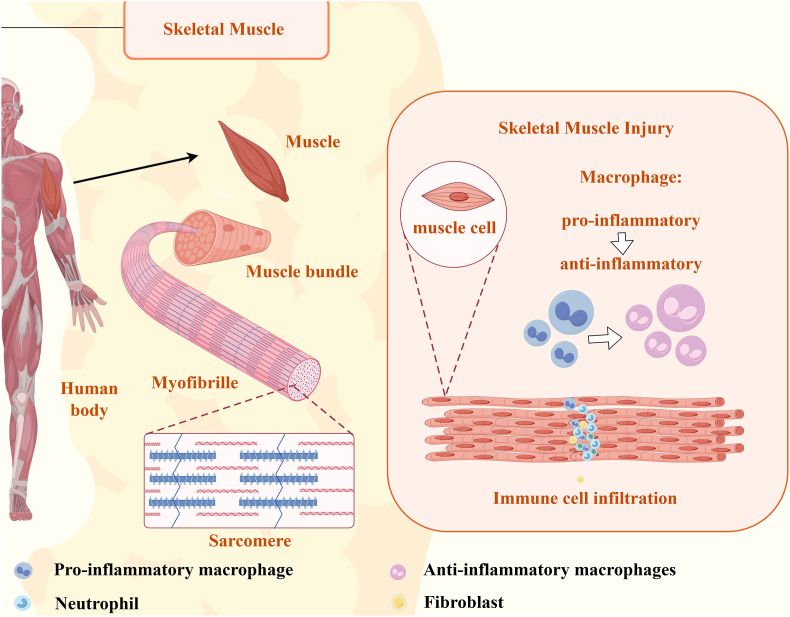
Schematic representation of skeletal muscle anatomy and the response to injury.

In response to muscle injury, the initial cellular reaction involves the infiltration of immune cells, particularly macrophages, which perform both immune and non-immune functions during the repair process [25]. These macrophages initiate an inflammatory response while also supporting muscle and bone mesenchymal stem cells (BMSCs). As the process progresses, pro-inflammatory macrophages shift to an anti-inflammatory phenotype, facilitating the resolution of inflammation and promoting tissue repair. The major repair processes in response to muscle injury consist of a destruction phase, a repair phase, and a remodeling phase. This multi-stage progression ensures that damaged tissue is cleared, regenerated, and structurally integrated [26]. Fig. 1 depicts the normal state of skeletal muscle and the regenerative process that occurs after injury.

Acute skeletal muscle injury is a common issue in sports-related trauma, and its treatment remains a focus of medical research. Current therapeutic approaches include pharmacological treatments, cell therapies, and physical rehabilitation, each with its limitations and challenges. Nonsteroidal anti-inflammatory drugs (NSAIDs), commonly used to alleviate pain and inflammation after acute muscle injury, may have adverse long-term effects on muscle recovery [27]. Other pharmacological treatments, such as anti-fibrotic agents like losartan, may even impair muscle function [28]. In terms of cell therapy, BMSCs, with the ability to differentiate into multiple lineages such as cartilage, bone, hematopoiesis-supportive stroma, and marrow adipocytes [29], are considered promising for muscle regeneration; however, studies indicate that while BMSCs survive post-injury, their impact on functional recovery is limited [30]. In physical therapy, functional electrical stimulation (FES) has been used to improve long-term neuromuscular function, although its efficacy varies among individuals [31]. Overall, the treatment of acute skeletal muscle injury continues to face significant challenges, including effective inflammation reduction, promotion of regeneration, and prevention of fibrosis.

Given the limitations of current therapeutic strategies for skeletal muscle injury—ranging from incomplete regeneration to potential side effects—there is an increasing need for innovative approaches that can address both functional recovery and tissue integration more effectively. In this context, nanotechnology has emerged as a promising avenue for advancing regenerative medicine. In particular, low-dimensional nanomaterials, especially 2D nanomaterials, offer unique physicochemical properties that closely mimic the structural and functional characteristics of native tissues. These materials not only enable precise cellular interactions at the nanoscale but also hold significant potential for enhancing drug delivery, modulating immune responses, and promoting tissue regeneration.

## 3 Low-dimensional nanomaterials and 2D nanomaterials

### 3.1 Nanomaterial and low-dimensional nanomaterials

The inherent complexity and precise organization of human physiological systems have driven contemporary biomedical research toward microscale and precision-oriented approaches for elucidating disease pathogenesis and therapeutic interventions. This scientific evolution has been synergistically accelerated by breakthroughs in materials science, particularly through the development of nanoscale components. Since the cellular response to nano-scaled patterns may precisely direct formation of appropriate functional tissue, it is generally required that bio-functional materials be designed to have structural similarity to native tissue in a nanoscale range [32]. These nanomaterials are widely used for drug delivery[[33], [34], [35], [36]], imaging [37], and disease therapy itself [38] during the past two decades.

The term “nano” means dwarf and nanotechnology can be understood as the multidisciplinary science of developing, characterizing, and synthesizing materials by controlling their sizes and shapes at the scale of nanometers [39]. This class of material has the characteristic that at least one of the dimensions lie between 1 and 100 nm [40]. They are noted for high surface-to-volume ratios, which enhances the properties of these materials substantially when compared to those of bulk materials. The dimensionality of a material is the major feature that discriminates against types of nanostructured materials [41]. Generally, nanomaterials are broadly classified in four different categories,(i) zero-dimensional(0D) nanomaterials (all the dimensions are in nanometer scale, e.g., nanoparticles), (ii) one-dimensional nanomaterials(1D) (any one of the three dimensions is of nanometer scale e.g., nanorods, nanowires etc.), (iii) 2D nanomaterials (any two of the three dimensions are of nanometer scale e.g., nanosheets, nanoplates, and nano-coatings) and (iv) three-dimension(3D) nanomaterials (three dimensions are larger than 100 nm and electrons are not confined in any direction) [42]. Among them, 0D to 2D nanomaterials are called low-dimensional nanomaterials.

### 3.2 2D nanomaterials

Among the emerging various innovative nanostructures, 2D nanomaterials have attracted great attention, thus becoming one of the hottest research topics in the field of nanotechnology. Based on the unusual mechanical strength, physicochemical properties (e.g., photonic, catalytic, magnetic, electronic properties, etc.) and multiple exciting functions, 2D nanomaterials have shown high potential for versatile applications ranging from the well-developed energy storage/conversion to emerging biomedicine [43]. Most 2D nanomaterials are single-or few-layer nanocrystalline materials with a planar morphology and almost all of the atoms exposed on the surface, which travel across the periodic table of elements including the category of transition metals, boron-/carbon-/nitrogen-/sulfur-group, etc.

Emerging from the groundbreaking isolation of graphene in 2004 [44], 2D nanomaterials has been gained far-reaching developments in multitudinous fields. In particular, since Liu et al. [45] for the first time discovered graphene as a robust nanocarrier for water-insoluble aromatic drug (SN38) delivery in 2008, 2D nanomaterials have been exponentially explored in versatile bio-applications, including the delivery of therapeutic agents, cancer therapy, biomedical imaging, biosensing and tissue engineering to overcome the intrinsic limits of conventional theranostic modalities.

When compared to 3D and 1D nanomaterials, 2D nanomaterials have the following advantages.

(I) A controllable thickness, which can be precisely controlled through various preparation methods, typically ranging from a few nanometers to tens of nanometers [46];
(II) A special electronic structure, including a regulated band gap, a distinctive Fermi surface, and a band structure [47].
(III) High specific surface area, which enhances their adsorption capacity and reactivity [48].
(IV) Unique optical and electrical properties, including exceptional light absorption, fluorescence, and Raman scattering [49].
(V) Controllable physical and chemical properties, such as band gap, electrical conductivity, electrochemical activity, etc.

2D nanomaterials are broadly categorized into inorganics and organics. The widely-used inorganics include:

(I) Transition metal dichalcogenides (TMDCs) (e.g., MoS_2_, WS_2_): TMDCs are semiconductors of the type MX2, where M is a transition metal atom (such as Mo or W) and X is a chalcogen atom (such as S, Se or Te). TMDCs exhibit a unique combination of atomic-scale thickness, direct bandgap, strong spin-orbit coupling and favourable electronic and mechanical properties, which make them interesting for applications in high-end electronics, energy harvesting, flexible electronics, DNA sequencing and personalized medicine [50,51].
(II) MXenes (e.g., Ti_3_C_2_): MXenes are a group of 2D carbides, nitrides, or carbonitrides of transition metals with layered structures and remarkable chemical properties and mechanical flexibility [52,53].
(III) Xenes: Xenes are a series of single-layer and monocomponent 2D nanomaterials ((X = Si, Ge, Sn and so on), which include graphene, black phosphorus etc. They are the most chemically tractable materials for synthetic exploration. Owing to their excellent physical, chemical, electronic and optical properties, Xenes have been regarded as promising agents for biosensors, bioimaging, therapeutic delivery, and theranostics, as well as in several other new bio-applications [54].
(IV) layered double hydroxides (LDHs) and clays: LDHs, so-called anionic clays, represent the class of lamellar compounds that are made up of cationic brucite-like layers with the intercalation of solvation molecules and exchangeable charge-balancing anions within the hydrate interlayer regions. Due to their high biocompatibility, pH-dependent bio-degradability and anion-exchange capacity, remarkable interest

has been focused on employing LDHs as desirable drug-delivery nanosystems for chemotherapy. Importantly, LDHs possess a wide range of metal-cation compositions, which could act as functional metal-cation carriers for antimicrobial and specific cancer theranostics [55].

Organic 2D nanomaterials encompass metal–organic frameworks (MOFs), COFs, and graphitic carbon nitride (g-C_3_N_4_), which offer porosity and modular functionality for drug loading and catalytic applications [56,56,57]. Table 1 presents a systematic classification of commonly studied 2D materials.

**Table 1:** Common classifications of 2D nanomaterials.

Material types	Material names	Descriptions	Examples
Inorganic 2D nanomaterial	TMDCs	Transition metal dichalcogenides with tunable electronic and optical properties.	MoX_2_ (X = S, Se, Te)
			WX_2_ (X = S, Se)
	MXenes	Transition metal carbides/nitrides with surface-terminated functional groups.	Ti_3_C_2_, Ta_4_C_3_, Nb_2_C
	Xenes	Monoelemental 2D nanomaterials	Graphene, Borophene, Black phosphorus
Organic 2D nanomaterial	LDHs and clays	Anionic clay materials composed of positively charged metal hydroxide layers intercalated with exchangeable anions.	Zn_2_/Al-LDH, Li/Al_2_-LDH, MOFs, COFs

Up to now, 2D nanomaterials have achieved impressive advances in biomedicine. For instance, a subset of 2D nanomaterials exhibit strong near-infrared (NIR) absorption, providing opportunities for photoacoustic (PA) imaging and photothermal therapy (PTT) at the first and/or second biological windows; some 2D nanomaterials can be utilized as efficient nanosensitizers (e.g. photosensitizers, sonosensitizers, radiosensitizers, etc.) for the production of reactive oxygen species (ROS) under the activation of alternative external physical activators (e.g., light, X-ray and ultrasound) or endogenous chemical energy triggers (e.g.,H2O2) for nanodynamic therapy (NDT) including photodynamic therapy (PDT), sonodynamic therapy (SDT), chemodynamic therapy (CDT) and radiodynamic therapy (RDT); and the high surface-to-volume ratios make 2D nanomaterial ideal for use as drug-delivery systems, on which they can load abundant guest molecules (e.g., fluorescent molecules, small-molecule drugs, genes and biomacromolecules) and show superior efficacy over controlled release where other functional moieties such as contrast agents can be integrated for achieving the purposes of specific disease treatment. These characteristics make them cutting-edge tools for a host of biomedical applications [58].

### 3.3 Preparation and synthesis of 2D nano-materials

2D nanomaterials have a layered structure, and the mobility of electrons is limited to nanometer lengths, which is comparable to being several atomic layers thick [59], exhibiting properties similar to those of graphene. When compared to three-dimensional (3D) and one-dimensional (1D) nanomaterials, 2D nanomaterials have the following advantages: (1) a controllable thickness, which can be precisely controlled through various preparation methods, typically ranging from a few nanometers to tens of nanometers [60]. (2) A special electronic structure, including a regulated band gap, a distinctive Fermi surface, and a band structure [61]. (3) High specific surface area, which enhances their adsorption capacity and reactivity [62]. (4) Unique optical and electrical properties, including exceptional light absorption, fluorescence, and Raman scattering [63]. (5) Controllable physical and chemical properties, such as band gap, electrical conductivity, electrochemical activity, etc. A major challenge in the current development of 2D nanomaterials lies in their synthesis and preparation. Two-dimensional nanomaterials can be synthesized by using physical and chemical methods, including mechanical exfoliation, ion intercalation, ultrasonic-assisted liquid phase exfoliation, chemical vapor deposition (CVD), and hydrothermal synthesis [64]. Moreover, the 2D nanomaterials exhibit different properties depending on their preparation conditions [65]. It is highly significant to investigate the cost-effective and efficient preparation of 2D nanomaterials and to enhance their functionality in the relevant application fields. Herein, this part provides an overview of the preparation methods for 2D nanomaterials and discusses the advantages and limitations of each method.

The preparation of 2D nanomaterials is of great significance for related research, with varying requirements for the surface morphology, lateral dimensions, and microstructure, depending on the intended application field. The preparation techniques for 2D nanomaterials have expanded with the progress of research and can now be broadly classified into “top-down” and “bottom-up” approaches [66]. The top-down preparation method involves the gradual removal of matrix material under an external force. The top-down approach relies on exfoliating thin layers of 2D crystals from their parent layered bulk crystals. It should be noted that top-down methods are only applicable to materials with layered compound bulk crystals. The main methods contains mechanical peeling, ultrasonic-assisted liquid phase exfoliation, and ion intercalation-assisted exfoliation [67]. The bottom-up approaches employed for the synthesis of ultrathin 2D nanomaterials involve controlled chemical reactions of specific precursors under well-defined experimental conditions. In principle, this bottom-up method offers greater versatility, enabling the potential fabrication of various types of ultrathin 2D nanomaterials. The main methods contains chemical vapor deposition (CVD), the hydrothermal method [68], physical vapor deposition (PVD), and atomic layer deposition (ALD), which can all be used to produce large-sized 2D nanomaterials with good microstructure.

In the fabrication of 2Dnanomaterials, top-down strategies, such as mechanical exfoliation, liquid phase exfoliation, and electrochemical exfoliation, employ external forces to disrupt van der Waals interactions, thereby facilitating the extraction of 2Dnanomaterials from bulk materials. Conversely, bottom-up approaches, such as CVD and solvothermal synthesis, synthesize 2Dnanomaterials directly from atomic or molecular precursors through chemical reactions. The choice of synthesis method markedly influences the properties of the resulting 2Dnanomaterials, with no single method universally ideal. Rather, each technique offers distinct advantages tailored to specific needs. For example, despite its limitations in yield and uniformity, mechanical exfoliation remains prevalent in research laboratories due to its simplicity and efficacy for small-scale studies. Future advancements are anticipated to optimize these methodologies, enhancing the efficiency, scalability, and environmental sustainability of 2D nanomaterial synthesis [69].

### 3.4 Nanomaterials for skeletal muscle

As mentioned before, the anatomical structure of skeletal muscle can be considered as a hierarchical complex with organized sub-micron sized self-contracting functional units, which imply an application of nano-sized materials for new therapeutic regeneration approaches. Also, communication between MuSCs and other components of the stem cell niche can be a significant target for biomedical engineering to improve the regenerative functions of skeletal muscle tissue throughout the entire muscle regeneration process [70]. Nanomaterials contains 0D-3D nanomaterials, 0d nanomaterials are rarely used alone. 1D nanomaterials are mostly used in skeletal muscle in the form of nanotubes and carbon nanotubes. In 2013, Martinelli et al. [71] discovered the ability of carbon nanotube scaffolds to promote cell division and maturation in cardiomyocytes. Zhao et al. [72] made a polyethylene glycol-linked multi-walled carbon nanotube (PEG-CNT) films to direct the skeletal myogenic differentiation of hMSCs in the absence of myogenic induction factors, which also suggests that the effect of a single dimension may be far less than that of two-dimensional and three-dimensional muscle repair. The application of 3D nano materials in skeletal muscle system is mainly applied to 3D bioprinting, which is manufactured by 3D printing Controllable assembly of skeletal muscle-like bundles [73]. In contrast, the application of two-dimensional materials in skeletal muscle system is more abundant and the prospect is more extensive. As we mentioned earlier, two-dimensional materials have a wide variety of physical and chemical properties which offer the promotion of cell migration and alignment, cell differentiation, biocompatibility, conductivity, and drug delivery properties. When combined with other bioengineering technologies, these distinct properties of 2D nanomaterials make it possible to fulfill unmet needs for the treatment of skeletal muscle disorders. The following sections will cover the concurrent application of 2D nanomaterials materials for skeletal muscle regeneration based on different treatment intention.

## 4 2D nanomaterials in skeletal muscle injury applications

### 4.1 Construction of biomimetic scaffolds

The regeneration of skeletal muscle mediated by muscle stem cells involves intricate cellular and molecular interactions, with satellite cells serving as the primary regenerative reservoir that activate upon injury, proliferate, and differentiate into myoblasts to reconstruct functional myofibers. However, pathological contexts such as muscular dystrophies or severe trauma often impair this regenerative cascade due to microenvironmental dysregulation [74]. Due to their unique physicochemical properties and biocompatibility, nanomaterials have shown great potential in muscle stem cell regeneration [75]. The construction of biomimetic scaffolds is one of the most well-known applications of 2D nanomaterials in skeletal muscle injury. Various types of 2D nanomaterials have been explored for this purpose.

**Fig. 2:**
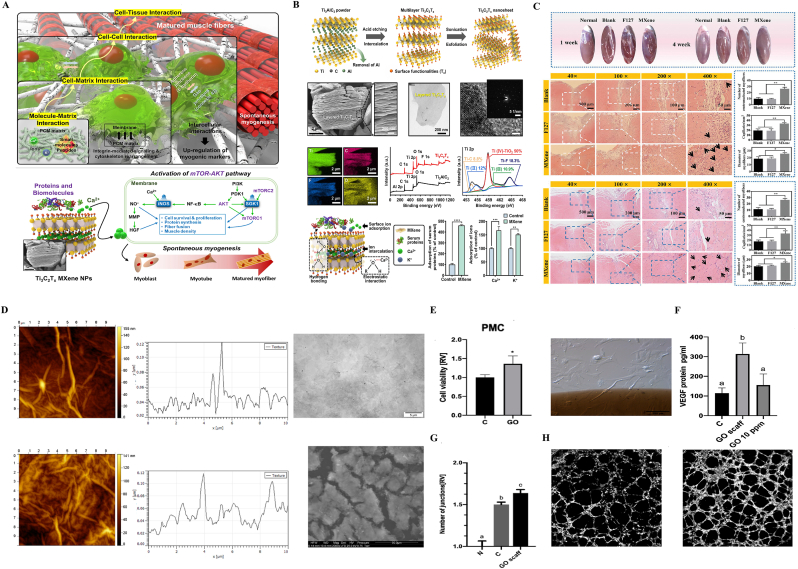
**Applications of 2D nanomaterials in construction of biomimetic scaffolds.** A) Ti_3_C_2_T_x_ MXene nanoparticles promote spontaneous myogenesis of C2C12 myoblasts by adsorbing proteins, biomolecules, and ions to activate the mTOR-AKT signaling pathway while supporting bottom-up sequential interactions on aligned nanofiber matrices. B) Synthesis and characterization of Ti_3_C_2_T_x_ MXene NPs. C) Histological evaluation of skeletal muscle regeneration after Ti_3_C_2_T_x_ MXene implantation in rat full-thickness muscle defect model in vivo. D**)** Graphene oxide scaffold morphology. **E)** Viability and morphologies of progenitor muscle cells from hind limb (PMC) on the GO scaffold. **F)** VEGF-A protein level in the cell medium after incubation of PMC. **G)** Graph showing the mean number of junctions of human umbilical vein endothelial cells (HUVEC) tubes in the field of view. **H)** Images of HUVEC tube formation in the control medium and with the post-incubation addition of the medium from the PMC cultured on a graphene oxide scaffold. A-B) Reproduced from Ref. [85]. Copyright 2024 The Authors. Published by Spring Nature. C) Reproduced from Ref. [86]. Copyright 2022, Wiley‐VCH GmbH. D-H) Reproduced from Ref. [87]. Copyright 2020 The Authors. Published by MDPI.

Several studies have demonstrated that Ti_3_C_2_T_x_ MXene nanoparticles (NPs) can be integrated into aligned nanofibrous matrices, forming a scaffold for muscle regeneration following VML (Fig. 2 A-B). [[76], [77], [78]]. The binding of Ca^2+^ to MXene nanoparticles triggered the activation of inducible nitric oxide synthase and the serum/glucocorticoid regulated kinase 1-mediated mTOR-AKT pathway [79], thereby promoting the differentiation and maturation of myoblasts. Moreover, Ti3C2Tx fibers can also function directly as artificial muscles with high muscle strokes and contractile work capacities. These fibers can be used in applications such as robotic arms, grippers [80] and even hold potential as a substitute for irreversible skeletal muscle injury.

Graphene has also been utilized as a scaffold for skeletal muscle regeneration due to its unique electrical conductivity. Studies have shown that C2C12 myoblasts exhibit higher cell adhesion and better spreading behavior on ultrathin thermally reduced graphene-based films compared to GO and glass slides [81], which results in the formation of highly aligned functional myotubes [82]. When electrical stimulation is applied, C2C12 myoblast differentiation into myotubes is accelerated on thermally reduced graphene substrates, with increased myotube length and cell coverage area. Gene expression levels related to cell contraction and myotube differentiation, such as α-actin, myogenin, myosin heavy chain isoform IId/x (MHC-IId/x), and sarcomeric actin, were significantly upregulated [81]. In a study by Mateusz et al. [83], using an embryonic chicken muscle development model, it was found that Graphene Oxide scaffold could simultaneously induce myogenic progenitor cell signaling pathways and activate proangiogenic signaling pathway by upregulating the exocrine secretion of vascular endothelial growth factor (VEGF) (Fig. 2 C-H). Due to these effects, the Graphene Oxide Scaffold served as a niche for myogenic progenitor cell colonization, promoting cell proliferation and acting as a suitable environment for their growth. The interactions between the scaffold and the myogenic progenitor cells further initiated muscle cell differentiation, contributing to the formation of new muscle tissue. Scaffold-based strategies leveraging 3D graphene architectures effectively mimic the structural and functional properties of native muscle tissue, facilitating stem cell differentiation and physiologically relevant muscle contraction [84], while graphene oxide scaffolds further stimulate myogenic progenitor cell differentiation and angiogenic activities through surface chemistry-mediated signaling pathways [83].

### 4.2 Cell recruitment

Researchers have identified a variety of 2D nanomaterials that play a significant role in cell recruitment, making them promising candidates for use in skeletal muscle regeneration following injury.

**Fig. 3:**
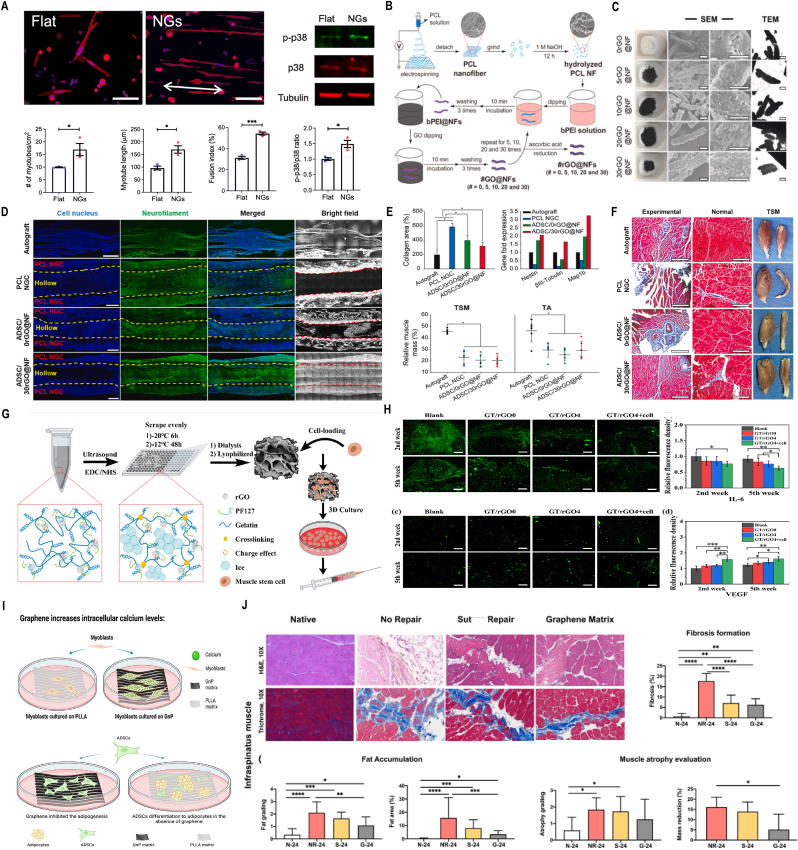
**Applications of 2D nanomaterials in muscle stem cell regeneration and differentiation. A)** Immunofluorescent images of myotubes stained for MHC showed that quantification of the relative number, length, and fusion index of myotubes formed by MPCs on nanograting substrates increased. Western blot analysis shows that p38 and phosphorylated p38 (p-p38) increased in young MPCs cultured on nanograting substrates. **B-C)** Schematic presentation and morphologies of graphene oxide (rGO)-modified polycaprolactone (PCL) nanofibers. PCL nanofiber is hydrolyzed in 1 M NaOH for 12 h and alternatively coated with bPEI and GO by electrostatic interaction. The coating is repeated 5, 10, 20 and 30 times to obtain different GO decoration amount. GO on nano fibers (NFs) is finally reduced by ascorbic acid to rGO for 48 h. **D-F)** Experimental results of the experimental leg and the normal leg, which demonstrated the potential of 2D nanomaterials in promoting muscle regeneration. **G)** The way to make the injectable conductive porous nanocomposite microcryogels based on gelatin (GT) and reduced graphene oxide (rGO). **H)** The immunofluorescence images of the regenerated muscle tissue after injection. **I-J)** Schematic illustration of the matrix in promoting robust myotube formation while concurrently inhibiting adipogenesis. A) Reproduced with permission [88]. Copyright 2023 The Authors. Published by American Chemical Society. B-F) Reproduced with permission [92]. Copyright 2023, Wiley-VCH. G-H) Reproduced with permission [93]. Copyright 2023, Wiley-VCH. I-J) Reproduced with permission [94]. Copyright 2023, Wiley-VCH.

Kai Wang and colleagues [88] discovered that 2D nanotopographical cues can promote myogenic differentiation of both primary and immortalized myoblasts. Compared to flat substrates, nanogratings enhanced the formation of myotubes, as evidenced by increased myotube number, length, and fusion index. nanogratings promoted the expression and phosphorylation of p38 mitogen-activated protein kinases (MAPK) (Fig. 3A). The integration of black phosphorus (BP) with conductive materials such as carbon nanotubes (CNTs) enhances the mechanical robustness and electrical conductivity of hydrogels, synergistically promoting the proliferation and differentiation of skeletal muscle cells through. Liu et al. [89] found that BP nanosheets embedded within hydrogels enable sustained release of phosphate ions, which act as bioactive signaling molecules to accelerate myogenic regeneration by activating AMPK/mTOR pathways. Notably, the combinatorial strategy of BP with stem cell-derived exosomes demonstrates amplified therapeutic efficacy, where exosomal miR-29b delivery cooperates with BP-mediated ionic modulation to suppress fibrotic markers while enhancing satellite cell recruitment and myotube fusion, establishing a pro-regenerative microenvironment [90,91]. Emerging research highlights the pivotal role of graphene in enhancing muscle regeneration, where rGO-modified polycaprolactone (PCL) nanofibers have demonstrated the capacity to promote neurogenic differentiation under electrical stimulation, significantly augmenting muscle mass and reducing collagen deposition in animal models(Fig. 3B-F) [92]. Advanced cell delivery systems, exemplified by gelatin methacryloyl/rGO (GT/rGO) microgels, exhibit the capability to preserve muscle stem cell viability during injection procedures and orchestrate localized regeneration at injury sites by suppressing collagen accumulation and inflammatory responses (Fig. 3 G-H) [93]. Notably, graphene nanoplatelet (GnP)-enriched matrices have shown dual functionality in promoting robust myotube formation while concurrently inhibiting adipogenesis (Fig. 3 I-J), thereby counteracting pathological muscle atrophy and ectopic lipid infiltration through mechanotransduction-mediated regulation of peroxisome proliferator-activated receptor-γ (PPARγ) signaling [94].

### 4.3 Drug delivery

2D nanomaterials are frequently utilized as drug delivery carriers due to their high loading capacity. In the context of skeletal muscle injury, a typical application involves utilizing 2D nanomaterials to carry therapeutic substances.

**Fig. 4:**
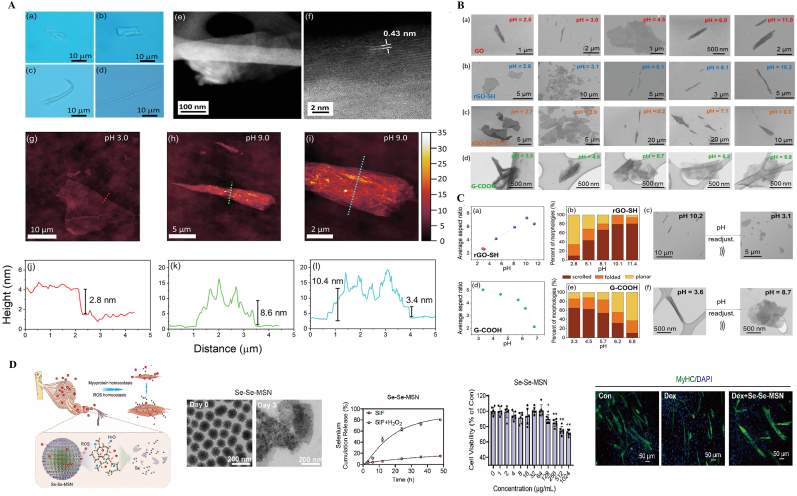
**Applications of 2D nanomaterials in drug delivery.** A) Characterization of different 2D electrolytes. B) Morphological configurations as a function of pH for different 2D electrolytes. C) Statistical analysis of 2D electrolytes: rGO-SH and G-COOH. D) ROS-scavenging Se–Se-MSNs restored Dex-induced dysregulation of myoproteins and redox homeostasis in vitro. A-C) Reproduced with permission [98]. Copyright 2021 The Authors. Published by Wiley‐VCH GmbH. D) Reproduced with permission [99]. Copyright 2024 Elsevier Ltd.

Siyue Tao et al. [95] developed a delivery platform based on two-dimensional MOFs, which enables targeted bone delivery of complement inhibitor-related therapeutics, along with pH-responsive controlled release. Although its efficacy has so far been validated primarily in patients with rheumatoid arthritis, skeletal muscle injury represents a promising next target for this approach. Additionally, some researchers have explored the functionalization of 2D nanomaterials such as graphene, graphene oxide (GO), reduced graphene oxide (rGO), and molybdenum disulfide (MoS_2_) by introducing various chemical groups [96], including oxygen-based groups (e.g., epoxy, carbonyl, hydroxyl, and carboxyl), trimethoxysilane (3-mercaptopropyl), and fluorescent molecules (e.g., fluorescein isothiocyanate isomer I, FITC). Upon dissociation in different solvents, these materials acquire a charge and function as 2D electrolytes. Under varying external conditions, such as changes in pH, temperature, ion concentration, and the dielectric constants of the medium, these 2D electrolytes exhibit reversible directional changes (Fig. 4 A-C). This enables them to respond rapidly to stimuli and facilitates targeted drug delivery to the injury site, offering a wide range of potential therapeutic applications. 2D nanomaterials have also recently been found to work synergistically with oral drugs to regulate myoprotein homeostasis and redox balance. Se-Se-MSNs loaded with leucine (Leu@Se-Se-MSNs) can serve as a supplement for the medical treatment of skeletal muscle loss (Fig. 4D) [97].

### 4.4 Photothermal therapy and antioxidant effects

PTT typically adopts the photothermal agent accumulated in diseased sites as the energy absorber for converting light to heat, inducing apoptosis and/or antiseptic and anti-inflammatory [100]. As a spatiotemporally controllable therapy approach, PTT has attracted considerable attention because of its low cost, excellent specificity and low side effects on normal tissues. Among all kinds of photothermal agents, 2D nanomaterials are influential candidate materials for PTT due to their unique properties and structure and black phosphorus is most commonly used in skeletal muscle repair.

As an in vitro application of GO, a stretchable and transparent muscle cell-graphene hybrid was fabricated by Kim et al. [101]. By utilizing electroconductivity and bio functionality of GO, researchers created a cell-sheet-graphene hybrid that enables recording, stimulation, and therapy in the skeletal muscle tissue. C2C12 seeded on the device showed improved elongation and alignment according to the electrical pulse stimulation. After implantation of the device in the hindlimb, optogenetic stimulation enabled muscle movements and implanted muscle cells proliferated on the muscle tissue.

Since black phosphorus quantum dots (BPQDs) and black phosphorus nanosheets (BPNSs) can effectively produce photothermal effects and ROS under near-infrared light irradiation, their stability can be improved through functionalized modifications (such as polydopamine modification or binding to fullerene C60), which can effectively kill bacteria and improve the inflammatory microenvironment, thereby promoting skeletal muscle regeneration.

On the other side, Ti3C2Tx MXene nanosheets are easily oxidized and prone to interlayer stacking, which will weaken their bioactivities, with the combination of polydopamine (PDA),they could steadly remodel microenvironment by eliminating ROS, decreasing the secretion of pro-inflammatory cytokine and upregulating autophagy to promote polarization of macrophages, vascularization and myogenic differentiation [102].

## 5 Existing challenges

Current studies primarily provide potential approaches to improve the inflammatory environment, promote stem cell differentiation, and facilitate muscle cell regeneration [103]. This limited application is closely related to the diverse nature and complex etiology of skeletal muscle injuries.

Furthermore, existing applications of 2D nanomaterials in skeletal muscle injury face several challenges, many of which are not unique to this field.

The primary concern is safety, or toxicity and biocompatibility. Factors such as material shape, size, chemical stability, surface modification, surface charge, and overall stability influence the toxicity of 2D nanomaterials [104]. At the same time, 2D nanomaterials can also undergo covalent modifications through various reactions, such as substitution reactions, free radical reactions, addition reactions, hydrolytic condensation reactions, and coordination reactions [105]. In addition, non-covalent interactions such as π-π stacking, electrostatic interactions, and hydrogen bonding can facilitate the attachment of organometallics (NOM), biomolecules, and synthetic compounds to the surfaces of 2D nanomaterials. These methods are used to regulate the fundamental properties of 2D nanomaterials, such as size, thickness, redox properties, cellular interaction sites, and colloidal stability, as well as integrated properties like dispersibility, biocompatibility, environmental stability, and conductivity, to meet different application needs. Changes in surface properties are crucial for nanomaterial-biointerface interactions, and alterations in electrochemical properties can significantly affect oxidative stress, ultimately influencing the toxicity of 2D nanomaterials. Moreover, surface modification and functionalization can also enhance biocompatibility. Functionalizing with biocompatible polymers or biomolecules helps reduce toxicity and immunogenicity. A thorough understanding of their physicochemical properties is essential before clinical application. The biological safety of different 2D nanomaterials may depend on various factors. For example, the biocompatibility of MXenes is influenced by factors such as concentration, particle size, and administration method [106].

In addition, due to the size effect of nanomaterials, unmodified nanoparticles may aggregate in vivo when their size is comparable to that of biomolecules, organelles, or cell membranes, potentially leading to persistent and cumulative adverse interactions with cells [107,108]. Their interactions with the immune system and potential effects on reproductive health also require further investigation. Moreover, there is currently no conclusive evidence confirming whether these 2D materials can be safely eliminated from the human body, raising concerns about their potential side effects. Extensive in vivo studies are essential to understand their pharmacokinetics, biodistribution, and long-term biological effects, including potential off-target effects, in order to ensure their safe and effective application in clinical settings.

Combining 2D nanomaterials with other materials has also emerged as a research hotspot. These composite systems expand the range of applications by integrating multifunctional capabilities such as hemostasis, anti-inflammation, antibacterial activity, tissue repair, and enhanced mechanical properties. However, this integration also introduces additional safety concerns. For instance, when 2D materials are not firmly bound to their carriers, powders or fragments may be released into tissues during wound care, potentially triggering immune responses and a cascade of pathophysiological processes. Additionally, the local tissue responses, biodegradation, angiogenesis, and nerve innervation that occur following material implantation can be attributed to the concept of skeletal interoception [109], offering new insights into the metabolic processes of 2D materials in vivo.

Systematic research is needed to clarify the relationship between the physicochemical properties of 2D nanomaterials and their biodegradability and biocompatibility. Moreover, most studies remain at the experimental stage, with significant barriers to clinical translation. Overcoming challenges related to toxicity, biocompatibility, stability, and biodegradation is crucial for future applications.

In addition, simplifying the synthesis processes of 2D materials and their composites, reducing costs, and improving the mechanical properties of these materials through chemical modifications and cross-linking with other substances, particularly for their use as scaffolds, will further facilitate the clinical translation of 2D nanomaterials and enhance their application value. Additive manufacturing, or 3D printing, has emerged as a foundational technology for bridging the gap between laboratory research and clinical application by enabling the layer-by-layer construction of complex 3D structures [110]. Beyond its structural capabilities, it also facilitates musculoskeletal regeneration by enhancing the interactions between bone and neural components [111]. It has already led to significant breakthroughs in musculoskeletal tissue repair and regeneration. In this context, although the clinical translation of 2D materials remains challenging, their potential remains highly promising. Addressing these issues requires deeper insights into the in vivo behavior of 2D materials, their interactions with biomolecules, long-term toxicity, and the precision control of their synthesis. Surface functionalization, as a solution to the toxicity of 2D nanomaterials, holds promising potential. However, it could inadvertently increase the complexity of synthesis and alter the inherent properties of the materials. Similarly, improving stability through modifications in synthesis techniques may affect other beneficial characteristics, such as biodegradability, potentially leading to unforeseen effects in vivo studies. To bridge the gap between experimental potential and clinical application, it is crucial to enhance in vivo research and foster interdisciplinary collaboration. This calls for interdisciplinary research integrating modern biomedical technology, physical chemistry, and precision manufacturing. For instance, organoids can be used to study the metabolism and biocompatibility of 2D materials. These 3D miniaturized human organs [112], derived from stem cell cultures, partially recapitulate the structural and functional complexity of real human organs, serving as transitional models for clinical applications.

## 6 Conclusion and perspective

**Fig. 5:**
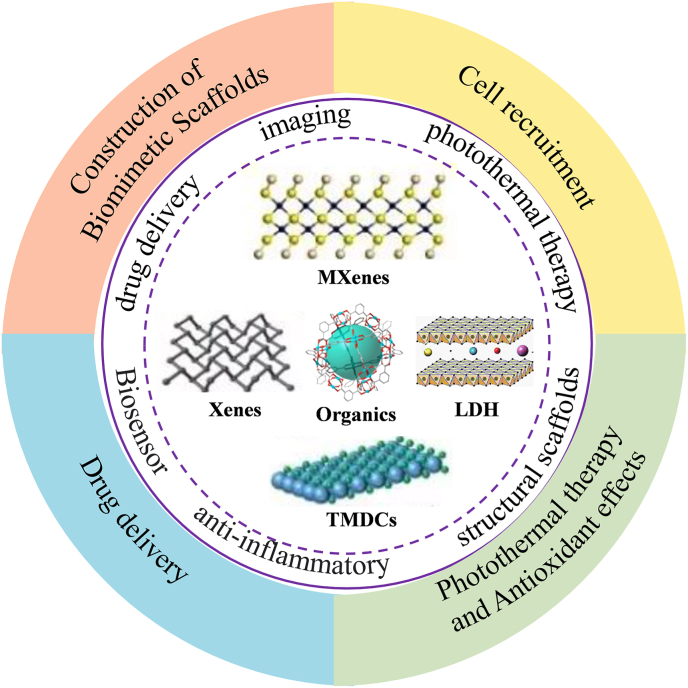
A schematic diagram of application of 2D nanomaterials in skeletal muscle.

With the increasing frequency and diversity of physical activities, skeletal muscle injuries resulting from overuse, improper posture, muscle imbalances, and muscle fatigue have become more prevalent. In addition, inevitable factors such as trauma, ischemic injury [113], severe infections [114,115], deep tissue injury [26], prolonged immobilization, and neurogenic muscle damage [116] can lead to muscle loss or even extensive muscle defects. These factors highlight the urgent need to address the repair and regeneration of skeletal muscle injuries. Fig. 5 shows a summary of the application of 2D nanomaterials in the skeletal muscle system.

As a prominent representative of low-dimensional materials, 2D nanomaterials possess high specific surface area, excellent biocompatibility, tunable mechanical properties, superior electrical conductivity, controllable biodegradability, and adjustable physicochemical characteristics [117,118]. These features enable them to align with and adapt to the physiological processes involved in muscle tissue repair and regeneration, making them highly effective in skeletal muscle injury. After summarizing recent research, we found that 2D nanomaterials, ranging from “signal agents” for new cell colonization to scaffolds for muscle regeneration, and from “accelerators” for stem cell differentiation to “inducers” of antimicrobial activity in inflammatory states, have demonstrated remarkable performance in the pathophysiological process of muscle injury.

Despite these advances, their clinical application in muscle injury remains in its early stages. Many 2D nanomaterials with significant potential have yet to be fully explored. In drug delivery, for instance, numerous strategies developed in oncology may be repurposed. LDH nanosheets can be loaded with anti-cancer drugs through electrostatic interactions [119]; MXene-based systems enable pH-responsive doxorubicin release, achieving an 82 % tumor inhibition rate in vivo [120,121]; and black phosphorus nanosheets allow 75 % mRNA knockdown of oncogenes like BCL-2 via high-capacity siRNA loading [122,123]. These amazing designs may serve as a promising drug delivery vehicle for skeletal muscle injury treatment by enabling controlled and sustained drug release at the injury site. In diagnostic imaging, the powerful contrast-enhancing capabilities of 2D nanomaterials remain underutilized for musculoskeletal assessment. Graphene quantum dots (GQDs) allow two-photon fluorescence imaging with a 1.2 mm tissue penetration depth, while WS_2_ nanosheets serve as dual-modal CT/MRI contrast agents with superior longitudinal relaxivity (r_1_ = 8.2 mM^−1^s^−1^) [124]. These compelling evidence collectively underscores the groundbreaking innovative potential of 2D nanomaterials in musculoskeletal imaging diagnostics.

Apart from that, PTT and PDT also hold potential for the clearance of necrotic tissues and cells in skeletal muscle injuries. For example, MXene materials, with their high specific surface area and photothermal efficiency, can convert light energy into heat, generating reactive oxygen species to induce tumor cell apoptosis [125]. MoS_2_@Au core-shell structures generate localized hyperthermia (55 °C under 808 nm laser irradiation), synergizing with chemotherapy for complete tumor ablation, whereas Ti_3_C_2_ MXene-mediated SDT enhances ROS generation by 3-fold via piezoelectric conversion of ultrasound energy [126,127]. These biomimetic design paradigm offers a transformative solution for combating infection-associated complications in musculoskeletal trauma rehabilitation, particularly the clinical challenges of deep-tissue antimicrobial therapy and hierarchical tissue regeneration.

Simultaneously, the immunomodulatory properties of 2D nanomaterials have witnessed remarkable progress in biomedical research, demonstrating tremendous potential for application in muscle injury repair. These atomically thin nanomaterials, including graphene derivatives and black phosphorus nanosheets, exhibit unique physicochemical properties that enable precise regulation of immune responses through multiple pathways: (1) Surface charge-mediated modulation of macrophage polarization toward anti-inflammatory M2 phenotypes, Specifically, LAPONITEs(a type of 2D nanoclays) significantly upregulated pro-inflammatory factors (IFN-g, TNF-a, IL-6, and IL-1b), downregulated anti-inflammatory factors (IL-10, IL-1ra, and Arg-1) and facilitated the phenotypic change from M2 to M1, whereas rBMSCs could reverse the polarization of RAW 264.7 cells from M1 to M2 and accelerate osteogenesis. It was confirmed that LAPONITEs assisted by BMSCs induced a mild immune response and improved bone regeneration in comparison with LAPONITEs alone both in vitro and in vivo [128]; (2) ROS-scavenging capability to mitigate oxidative stress in immune cells (3) Controlled release of bioactive ions (e.g., Mg^2+^, PO_4_^3−^); to orchestrate cytokine expression profiles [[129], [130], [131]]. Such capabilities allow integration of immunomodulation with regenerative processes, offering composite strategies for more effective muscle injury repair.

We also find that for various forms of skeletal muscle injury, such as strains (caused by overstretching or excessive contraction, leading to microtears in muscle fibers), contusions (resulting from external impact without complete fiber rupture, but often accompanied by edema due to blood vessel damage), muscle spasms (characterized by sudden, involuntary muscle contractions), tendinitis, and muscle fibrosis, the application of 2D nanomaterials has not yet been specifically optimized for distinct injury types. Aside from a specific type of skeletal muscle injury, VML, which is defined as the loss of at least 20 % [132] of muscle mass and typically occurs following traumatic events, targeted 2D scaffolds [133] have been developed to enhance clinical outcomes. Moreover, the repair of injured skeletal muscle is a complex process involving the coordinated actions of immune cells, muscle cells, perivascular cells, and neural components [134,135].

The multifunctionality and intelligent properties of 2D nanomaterials represent key advancements in their applications. By integrating their physicochemical, biological, and electrical characteristics, these materials can respond to external stimuli and be customized to match diverse injury types. Through the rational design of 2D nanomaterials, combined with computational modeling and a comprehensive understanding of their physical, chemical, and structural properties, it is possible to develop a new generation of adaptive and programmable intelligent structures. These advanced materials can be tailored for various types and degrees of skeletal muscle injury repair and regeneration, meeting diverse therapeutic needs. It is worth noting that a carbon-based artificial muscle, hydrogen-substituted graphdiyne muscle (HsGDY-M) was developed in 2025 to enable intelligent task performance, marking a promising step toward the future intelligent design and application of 2D nanomaterials.

In recent years, 2D nanomaterials have found extensive applications in biomedical fields such as drug delivery, biosensing, bioimaging, and regenerative medicine. This review outlines key members of the 2DNM family, including TMDCs, MXenes, and LDHs, and highlights their strategic advantages and pioneering contributions to skeletal muscle injury repair. However, several inherent challenges remain in the application of 2DNMs, including the varying synthesis requirements for different applications, the complexity of interactions between bioactive substances loaded onto 2D nanomaterials (such as drug loading efficiency, non-specific binding, aggregation, and potential loss of bioactivity from bench to bedside), and potential toxicity concerns. Additionally, the differences between external environments and the internal human body microenvironment can affect the performance of 2D nanomaterials in clinical applications. As in other emerging biomedical fields, 2D nanomaterials face both significant opportunities and technical challenges. With continued exploration in regenerative medicine, their unique properties are expected to align more closely with muscle tissue pathophysiology and integrate with the adjacent skeletal and neurovascular coupling [136], ensuring both precision and stability while maintaining excellent biocompatibility. These advances hold promise for more effective, durable treatments for skeletal muscle injuries.

## CRediT authorship contribution statement

**Hongyu Bai:** Writing – original draft, Visualization, Investigation. **Lu Liu:** Writing – original draft, Visualization, Investigation. **Zhiwen Luo:** Writing – review & editing, Conceptualization. **Renwen Wan:** Writing – review & editing, Conceptualization. **Jiwu Chen:** Writing – review & editing, Supervision.

## Availability of data and materials

Not applicable.

## Funding sources

This review did not receive any specific grant from funding agencies in the public, commercial, or not-for-profit sectors.

## Declaration of competing interest

The authors declare that they have no known competing financial interests or personal relationships that could have appeared to influence the work reported in this paper.

## Data Availability

No data was used for the research described in the article.
